# New evidence for rice harvesting in the early Neolithic Lower Yangtze River, China

**DOI:** 10.1371/journal.pone.0278200

**Published:** 2022-12-07

**Authors:** Jiajing Wang, Jiangping Zhu, Dongrong Lei, Leping Jiang

**Affiliations:** 1 Department of Anthropology, Dartmouth College, Hanover, New Hampshire, United States of America; 2 Pujiang Museum, Zhejiang, People’s Republic of China; 3 Longyou Museum, Zhejiang, People’s Republic of China; 4 Zhejiang Provincial Institute of Cultural Relics and Archaeology, Zhejiang, People’s Republic of China; 5 Department of History of Science and Scientific Archaeology, University of Science and Technology of China, Anhui, People’s Republic of China; University of California Santa Cruz, UNITED STATES

## Abstract

The Lower Yangtze River of China has been identified as an independent center of rice domestication, but tracing the earliest evidence for rice cultivation practices has been challenging. Here we report the first evidence for rice harvesting, based on use-wear and phytolith residue analyses of 52 flaked stone tools (10000–7000 BP) from the Shangshan and Hehuashan sites. The tools reflect two harvesting methods: reaping the panicles at the top and cutting the stalk near the base. Thus, our research provides a new method for investigating prehistoric cereal cultivation, and the data lend support to the evidence of rice domestication in the early Holocene. The results also show the complexity of rice harvesting strategies several millennia before the emergence of full-fledged agriculture in the Lower Yangtze.

## Introduction

The origin of rice agriculture in the Lower Yangtze River of China brought profound environmental and cultural transformations. Recent research indicates that rice domestication was a protracted process, which began in the early Holocene Shangshan culture (10000–8200 cal. BP), progressed continuously for several millennia, and culminated in the Liangzhu culture (5300–4400 cal. BP) [1–4]. Most studies have focused on analyzing morphological changes of rice remains to trace the timeline of rice domestication, whereas few scholars have paid attention to the cultivation practices that gave rise to domesticated forms. This paper investigates the role of harvesting in rice cultivation, and its relation to the prolonged development of rice farming.

Harvesting functions as a selective agent in plant domestication [5]. In most cereal crops, the loss of seed shattering is the hallmark of domestication, which renders a plant dependent on humans for propagation [6]. Archaeologists have long attributed this to the early use of harvesting sickles, a technique that selects for seeds with tough rachis, present in a small proportion among the wild population of cereals [5, 7, 8]. This model has gained increasing support from the Fertile Crescent, where large numbers of flint-edged sickles have been identified at pre- and early Neolithic sites [9–12]. In the Lower Yangtze, however, typical harvesting tools have not been reported from early Neolithic sites. Some scholars have thus proposed that sickle harvesting did not drive the initial stages of rice domestication; alternative methods, such as hand plucking and beating, may have been used [13, 14]. These conclusions were based on seed morphology and statistical modeling but lacked direct archaeological evidence.

Recent excavations from the Shangshan (10000–8200 BP) and Kuahuqiao (8000–7000 BP) culture settlements have recovered large quantities of flaked stone tools, which have not received much attention from archaeologists investigating the origins of agriculture. These tools, following the core-and-flake tradition in southern China, appear crudely fashioned and expediently made [15]. In the excavation reports, they are categorized as “scrapers”, “burins,” and “drills” based on their shape and edge characteristics [16, 17]. However, many of them exhibit acute edges that would have been suitable for harvesting plants. Our hypothesis was that some of the flaked tools were used to harvest plants including rice. To test this hypothesis, we conducted use-wear and phytolith residue analyses on 52 flaked tools.

Cereal harvesting produces distinctive use-wear traces and plant tissue residues on the tool surface. Experimental studies show that harvesting Poaceae plants (e.g., cereals, reeds, and cattails) produce particular use-polish and striations on a tool’s working edge and the pattern is generally similar across a variety of East Asian raw materials [18–21]. At the same time, harvesting plant accrues residues from plant seeds, stems, and leaves on the tool surface, providing more refined information regarding the particular plant types and parts being harvested [22–24]. Phytolith analysis is particularly useful for identifying rice harvesting tools because rice produces three diagnostic morphotypes: double-peak husk cells, *Oryza*-type bulliform leaf cells, and scooped parallel bilobates (typical of the Oryzeae tribe). By comparing the results from use-wear and residue analyses, we can obtain more reliable information to identify tool functions [25].

## Archaeological background

Shangshan and Kuahuqiao were the two earliest Neolithic culture groups in the Lower Yangtze Valley. The Shangshan culture people were the first in the region to engage in rice cultivation and sedentism [1, 26]. Recent archaeological investigations have identified 19 settlements, many of which have yielded rice seed and phytolith remains showing evidence of early-stage rice domestication [1, 26, 27]. The subsequent Kuahuqiao culture shows clear cultural continuities from Shangshan, with evidence of intensified landscape modifications and specialized earth-working tools [17, 28].

**Fig 1:**
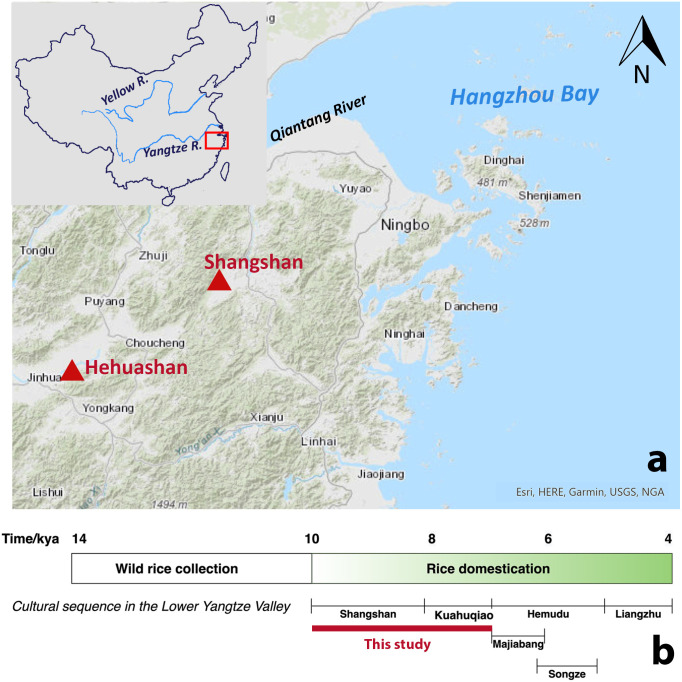
Archaeological background. (a) Location of Shangshan and Hehuashan, base map modified from USGS National Map Viewer (http://viewer.nationalmap.gov/viewer/); (b) Cultural history and rice domestication process in the Lower Yangtze River region.

The flaked stone tools examined in this study were recovered from two sites located in the Jinqu Basin of the Zhejiang Province: Shangshan—the type-site of the Shangshan culture—and Hehuashan—a settlement that was occupied by Shangshan and Kuahuqiao cultures (Fig 1). Archaeological surveys and excavations at Shangshan have identified an early Neolithic village approximately 30000 m^2^ in size, consisting of houses, trash and storage pits, and possible burials. The lithic assemblage is dominated by flaked stone tools (82%) and grinding stones (15%) with a small proportion of polished stone tools (3%) [16]. The Hehuashan site is located about 80 km southwest of Shangshan and lies on a small hill in Longyou County. The settlement is divided into two areas, East and West. The East area (600 m^2^ excavated) was occupied by the Shangshan culture (ca. 10000–8200 cal. BP), and contained houses, pits, and Shangshan style lithic and ceramic artifacts; the West area dates to the Kuahuqiao culture, and a small-scale excavation of 150 m^2^ area uncovered pits, ditches, houses, and Kuahuqiao style ceramics and stone tools [29].

Chipped flakes constitute a major component of the lithic assemblages at Shangshan and Hehuashan (82% and 57%, respectively). At the macroscopic level, the flakes appear expediently made and casually discarded: most of them do not exhibit retouch and are recovered from various archaeological contexts. Their forms and edge characteristics show clear differences from the hafted sickle blades from the Near East or the denticulate-edged sickles from the Huai River Valley of China [20, 30, 31]. They were made of local materials that included vitric tuff, river pebble, porphyry, and sandstone; no clear technological change from the Shangshan to Kuahuqiao periods has been observed [16, 29].

## Materials and methods

**Fig 2:**
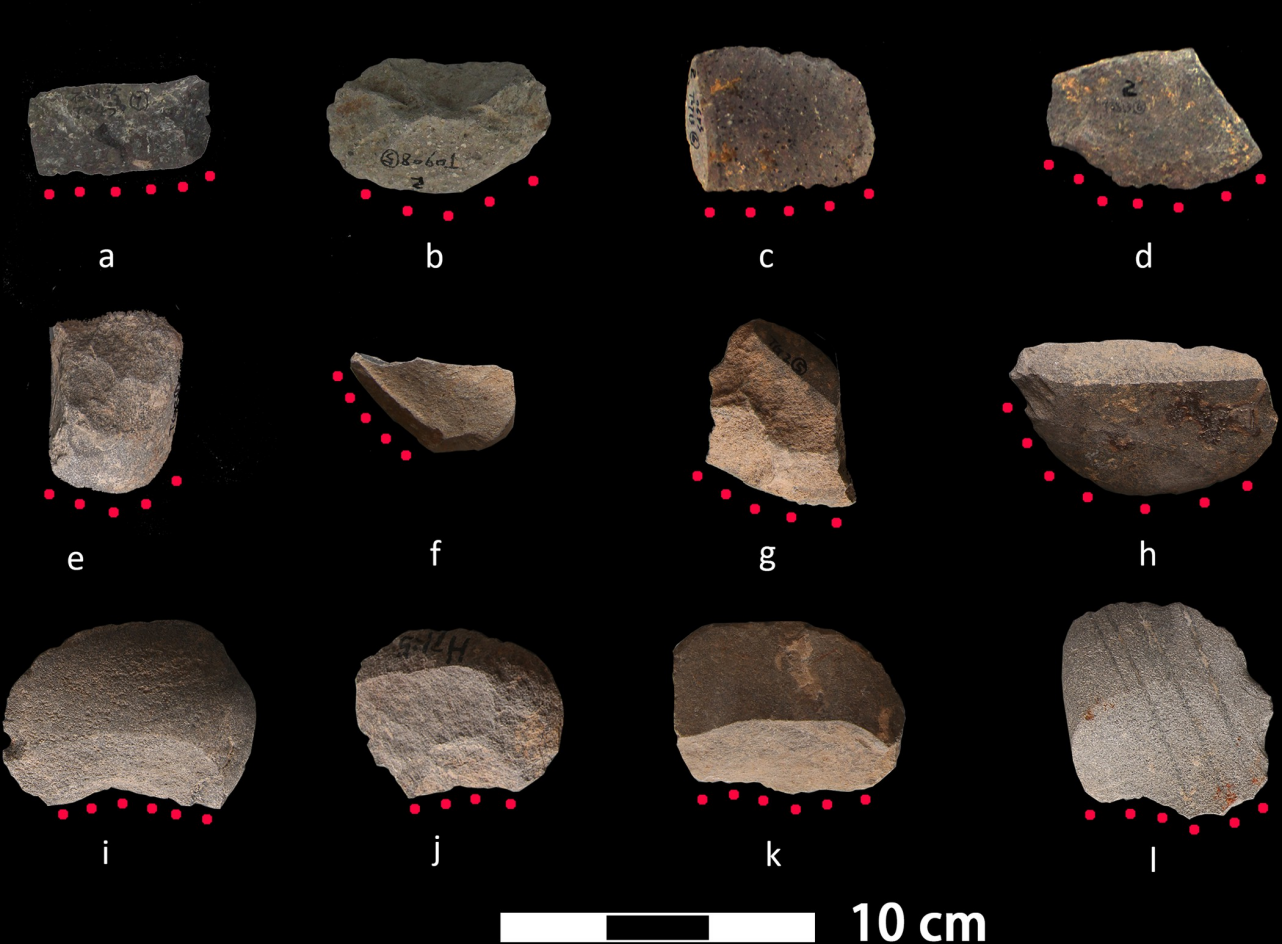
A selection of stone flakes analyzed in this study. (a)-(h) Flakes from the Shangshan culture; (i)-(l) Flakes from the Kuahuqiao culture. Red dots delineate working edges.

The flaked tools examined in this study were curated in the storage facilities of Pujiang Museum and Longyou Museum, where field sampling took place. No permits were required for the described study, which complied with all relevant regulations. Using a Dino-Lite microscope (10× to 50×), we selected 52 specimens that show mechanical edge modifications including rounding, polish, and retouch (Fig 2). These include 20 flakes from the early Shangshan phase (ca. 10000–9000 BP), 18 from the late Shangshan phase (ca. 9000–8200 BP), and 14 from the Kuahuqiao culture (ca. 8000–7000 BP). Given their shape and small sizes (average width = 42. 8 mm, average length = 42 mm; STDEV = 17.1 mm), the flakes are most likely handheld tools.

The procedure for residue sampling followed two steps. First, all artifacts were washed in distilled running water for 1 minute. This step helped to remove the contamination from surface sediments and the curation process. After washing, the artifacts were subject to sonication for residue extraction. Each specimen was placed in a new, polyvinyl bag with 15 mL of distilled water. The bag was then placed into the sonication bath for 6 minutes. After 6 minutes, the artifact was removed from the bag.

Laboratory processing of phytolith residue samples followed the established protocol [32]. Phytolith residue slides were scanned using either a Zeiss Axioscope A1 microscope or a Zeiss Axioscope 5 microscope at 400× magnification, both fitted with polarizing filters and DIC optics. Phytoliths were counted on entire slides and described using the International Code for Phytolith Nomenclature 1.0 [33]. Their identification was based on a reference collection from over 1200 Asian economically important plant specimens in the Stanford Archaeology Center.

Use-wear samples were taken from flakes after residue extraction. Polyvinyl siloxane (PVS) compound peel (President Light Body) was applied to the flake edges to provide a portable and durable record for microscopic analysis. The use-wear peels were examined under a Zeiss Imager. A2M microscope at magnifications of 50x, 100x, 200x, and 500x. Use-wear interpretations were based on a comparison with the reference data generated from a series of experimental studies conducted by the authors and colleagues [18, 19]. These experiments include harvesting Poaceae plants (cereals, reeds, and cattails; cutting stems and seed heads), butchery (carcass skinning, cutting animal tissue), and scraping bones, tree branches, and bamboo. Two types of tool motions were used in the experiments: longitudinal and transverse. Longitudinal motions include slicing and sawing (i.e., unidirectional or bidirectional and parallel to the edge); transverse motion includes scraping, pulling, and whittling.

The experimental studies provide two findings particularly relevant to this study. First, cutting and processing Poaceae plants produce use-wear traces similar across raw material types common in China, including for example, sandstone, tuff, and quartz. The wear traces are characterized by fine striations, high polish, and rounded edges on crystal grains, which can be separated from those from working on other materials, such as wood, animal tissues, and bones. Second, the characteristics of striations can be used to infer the mode by which tools are used. Harvesting with a slicing motion near the plant base produces striations parallel to the cutting edge, whereas harvesting by cutting at the plant top generally involves a transverse action, creating striations parallel or diagonal to the cutting edge. Therefore, the use-wear reference collection allows us to identify not only plant harvesting tools but also harvesting methods.

## Results

### Use-wear analysis results

Use-wear analysis indicates that the flakes have been used for five types of tasks, including harvesting siliceous plants (N = 30), cutting animal tissues (N = 7), processing hard materials (N = 10), scraping woody materials (N = 6), and unidentified tasks (N = 13). The frequency used here represents functional counts of working edges rather than tool quantity because 14 tools have two working edges. Use-wear characteristics for individual flakes are summarized in the S1 Table. We describe the main characteristics of the four identified functions below.

#### Harvesting siliceous plants

**Fig 3:**
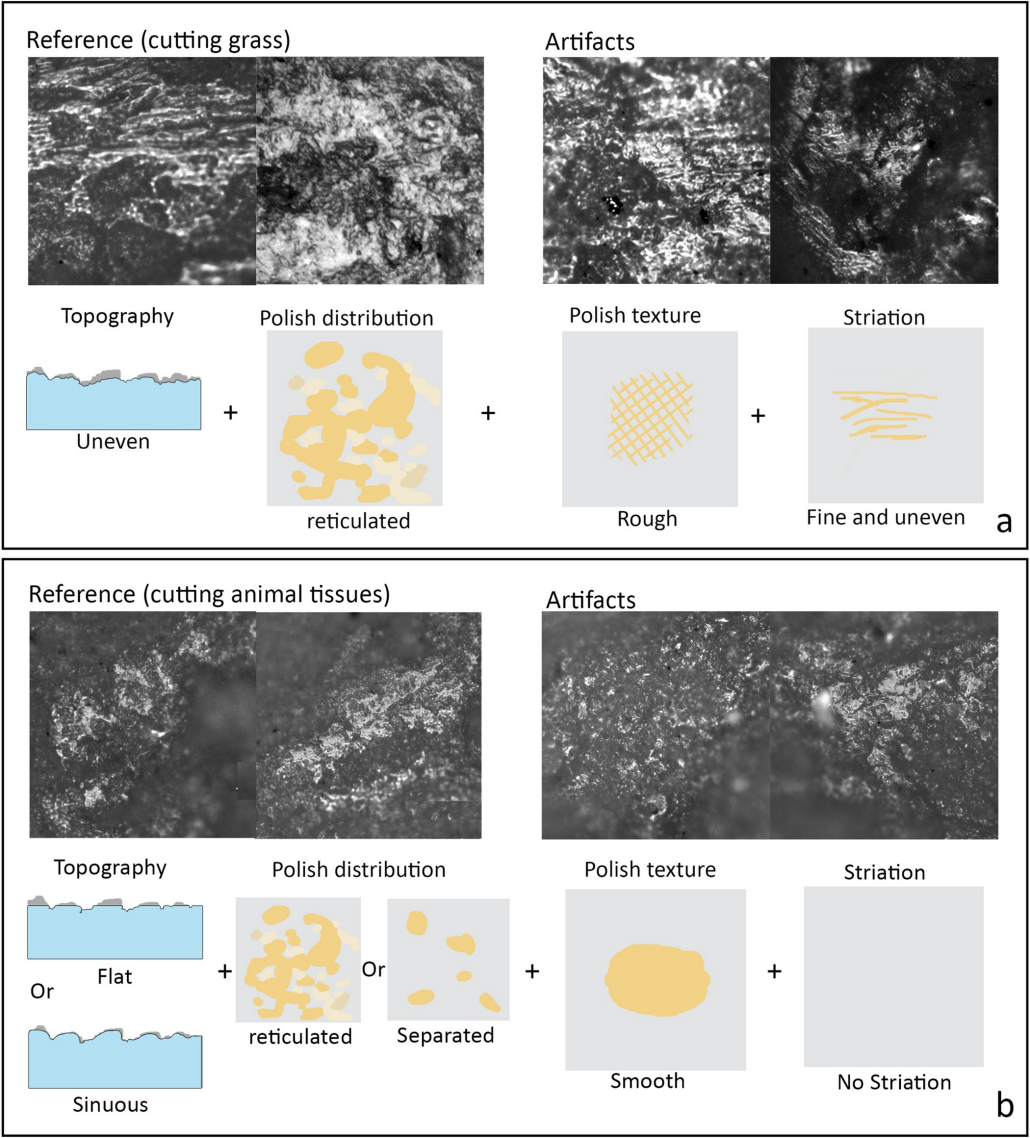
Use-wear traces from cutting grass (a) and cutting animal tissues (b).

(Fig 3A). Thirty flakes show use-wear patterns consistent with cutting soft plants, such as grasses and reeds. Their cutting edges generally show an uneven and rough topography, extensive and reticulated polish that distributes on both topographic highs and in the interstices, and fine and uneven striations. Among these, 22 tools show striations oriented predominantly perpendicular or diagonal to the working edge, indicating a transverse action such as cutting and scraping. Fourteen tools exhibit striations running parallel to the cutting edge, likely a result of slicing activities. Six tools show both types of striation alignments.

#### Cutting animal soft tissue

(Fig 3B). Six flakes show use-wear patterns related to cutting animal tissues, suggesting that they were used as butchering tools. Unlike the rough surface of plant cutting flakes, cutting soft animal tissues builds up smooth and sporadic patches of polish without striations on a sinuous or leveled topography.

#### Scraping woody materials

**Fig 4:**
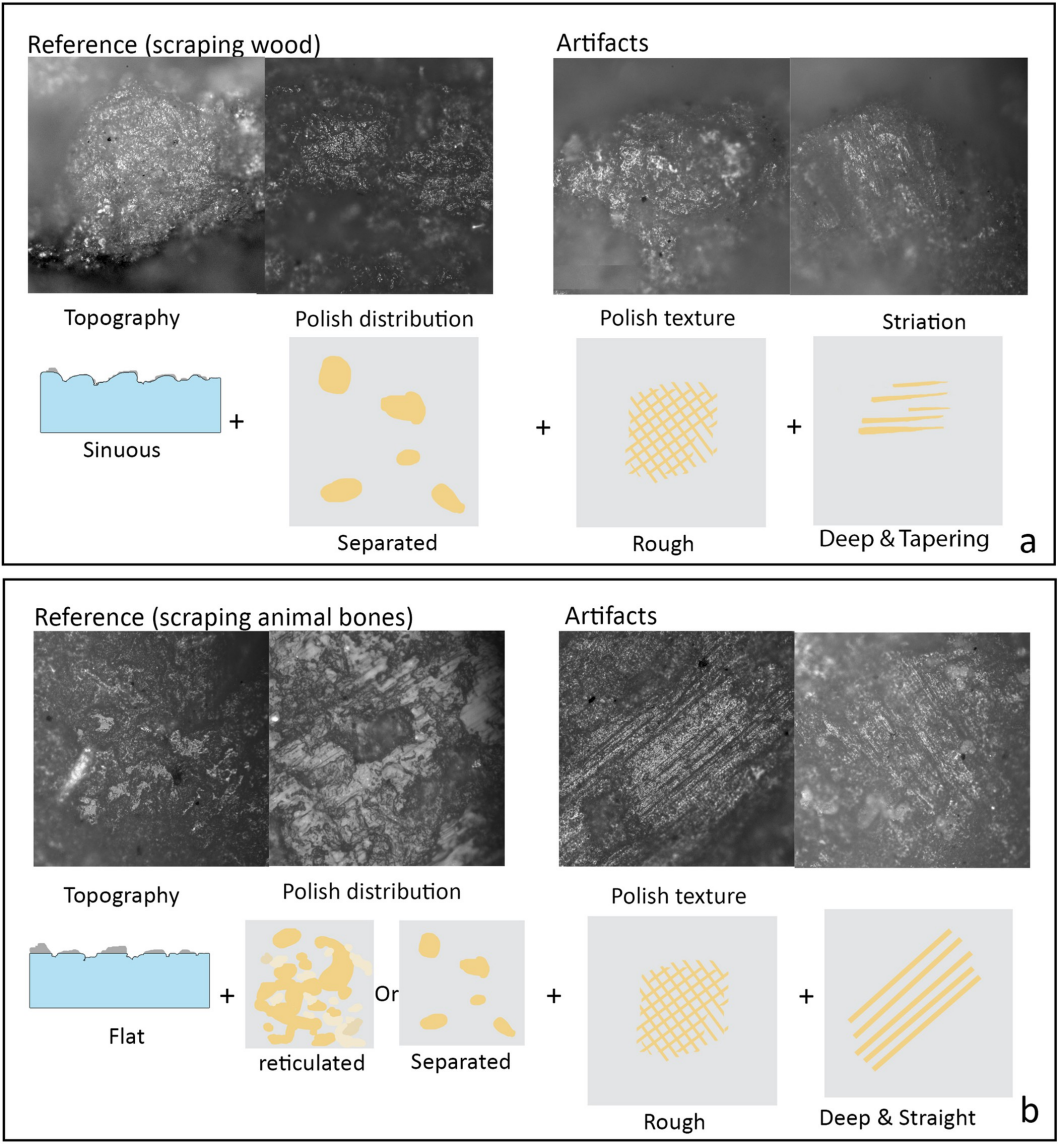
Use-wear traces from scraping wood (a) and scraping animal bones (b).

(Fig 4A). Six flakes are identified as wood scraping tools. Their use-wear shows rough polish on a sinuous or “domed” topography, distributed in separated patches. Striations are occasionally present, which are deep and tapering, running perpendicular to the working edges.

#### Scraping hard materials

(Fig 4B). Ten flakes show distinctive use-wear traces consistent with those produced by scraping hard materials, such as animal bones. They show a leveled topography and rough polish. Striations are deep, straight and even, which can be distinguished from those caused by softer materials, such as Poaceae plants and woody materials. Polishes are concentrated on high topography, either in separated or reticulated patches.

One interesting finding from our use-wear analysis is that some flakes are multifunctional. This pattern is frequently observed on tools with multiple working edges. For example, Artifact HHS 21 has two working edges, one concave with a sharp edge angle (15°), and the other convex with a larger edge angle (45°– 60°). The concave edge exhibits wear patterns consistent with processing siliceous plants, whereas the convex edge has traces similar to those from processing animal tissues, possibly including hard materials like bone. Thirteen flakes in total with multiple working edges show evidence of multifunctional use.

### Phytolith analysis results

**Fig 5:**
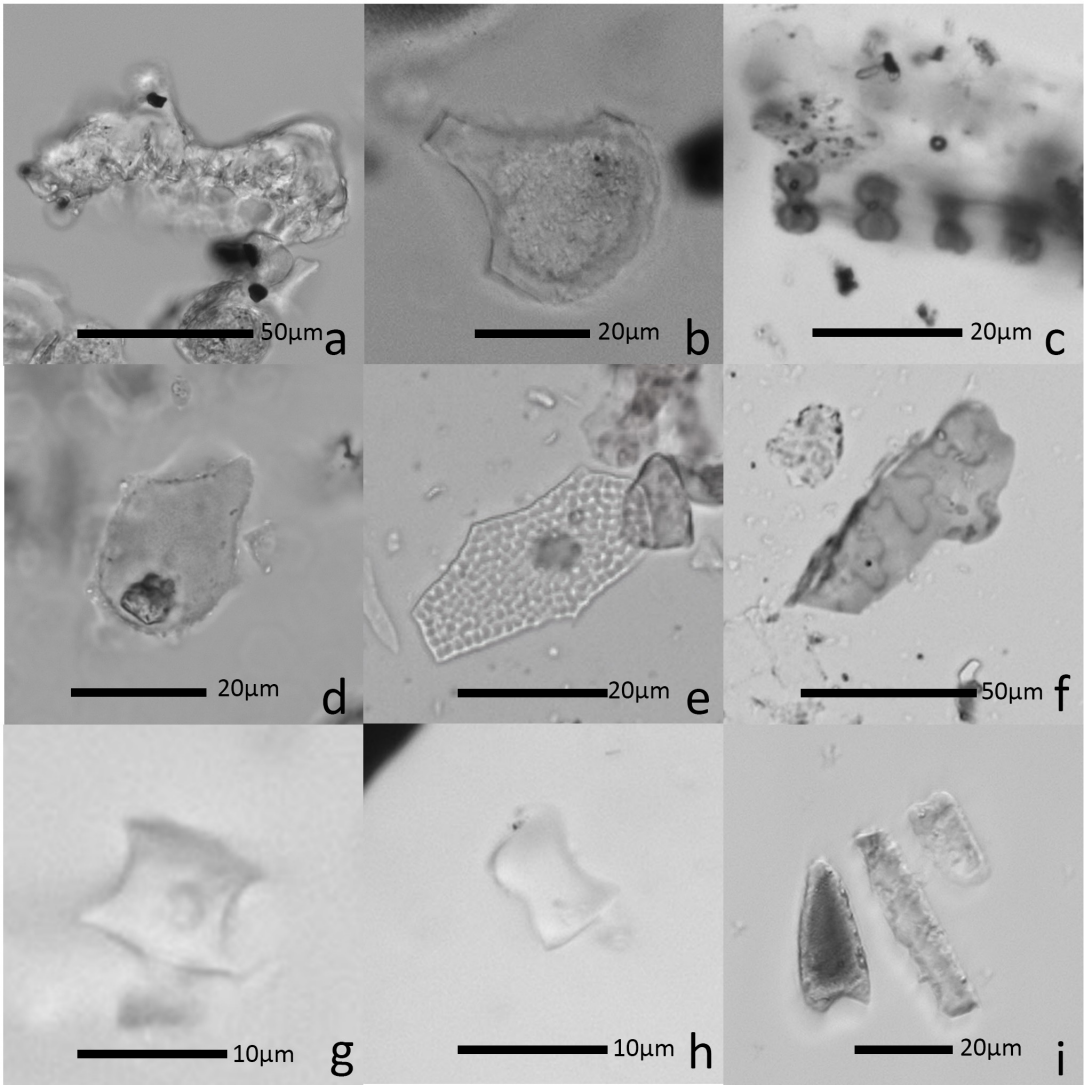
Phytolith morphotypes recovered from flakes from Shangshan and Hehuashan flakes. (a) Double-peak (*Oryza*, rice); (b) *Oryza*-type bulliform (*Oryza*, rice); (c) Scooped parallel bilobate (Oryzeae); (d) Reed-type bulliform (*Phragmites*); (e) Sedge achene (Cyperaceae); (f) Articulated quadrilobate (Panicoideae); (g) Rondel (Poaceae); (h) Saddle (Poaceae); (i) Scutiform, elongate sinuate, and rectangular (Poaceae).

Phytoliths were found on 50 of 52 flakes and their quantities varied considerably (S2 Table; MIN = 0, MAX = 241, STDEV = 69). The majority of the phytolith types belong to grass species (Poaceae), dominated by psilate or sinuate elongates from grass leaves, stems, and inflorescences (Fig 5). More diagnostic morphotypes include double-peak (*Oryza* sp., rice) (Fig 5A), *Oryza*-type bulliform (*Oryza* sp., rice) (Fig 5B), parallel scooped bilobate (Oryzeae) (Fig 5C), reed-type bulliform (*Phragmites* sp.) (Fig 5D), sedge achene (Cyperaceae) (Fig 5E), and articulated quadrilobate (Panicoideae) (Fig 5F). Overall, our phytolith data indicate that a high proportion of flakes was used for harvesting siliceous plants.

**Fig 6:**
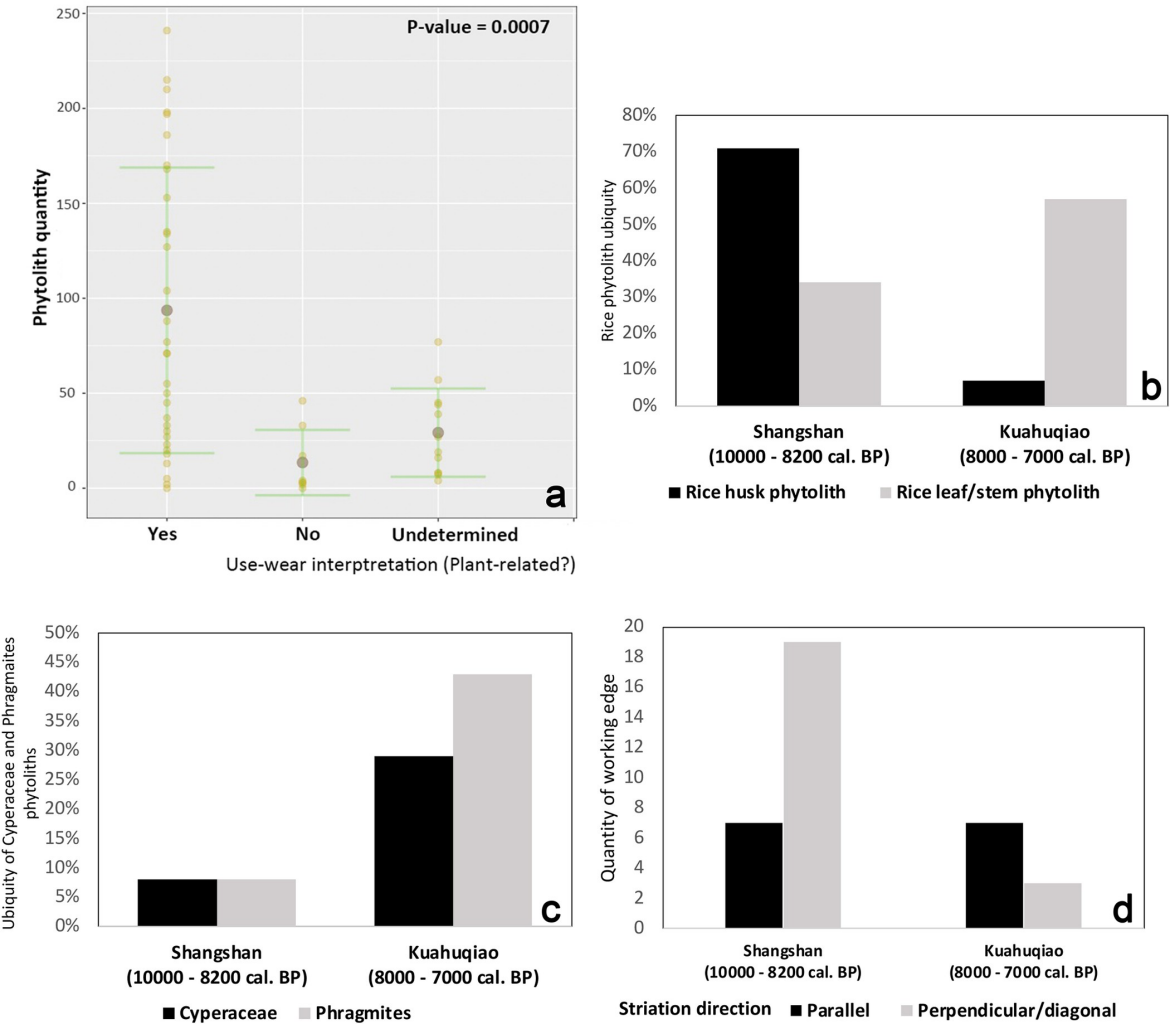
Statistical analyses of phytoliths and use-wear patterns from the Shangshan and Kuahuqiao flakes. (a) Results of Kruskal-Wallis analysis comparing phytolith richness and use-wear interpretation; (b) Rice phytoliths ubiquities; (c) Cyperaceae and *Phragmites* phytoliths ubiquities; (d) Use-wear striation alignments from the flakes with wear traces related to plant harvesting.

A comparison of the phytolith assemblages from Shangshan and Kuahuqiao culture flakes shows two major differences (Fig 6). First, the ubiquity of rice double-peak husk phytolith decreases significantly from 71% to 7%, whereas the ubiquity of rice leaf/stem phytoliths increases from 34% to 57% (Fig 6B). Over the same period, phytoliths of sedges and reeds become more ubiquitous, increasing from 8% to 29% and 43%, respectively (Fig 6C). These differences may be associated with shifting harvesting techniques and landscape management practices (See Discussion and Conclusion).

### Integrating use-wear and phytolith results

We conducted a Kruskal-Wallis test to compare the results of use-wear and phytolith analyses. The analysis shows a strong correlation between phytolith richness and use-wear interpretation (Fig 6A). The tools showing plant-related wear traces contain significantly higher quantities of phytoliths than those for other tasks (P-value = 0.0007). Overall, the results of use-wear and phytolith analysis corroborate each other, indicating that a high proportion of flakes have been used to harvest siliceous plants.

**Fig 7:**
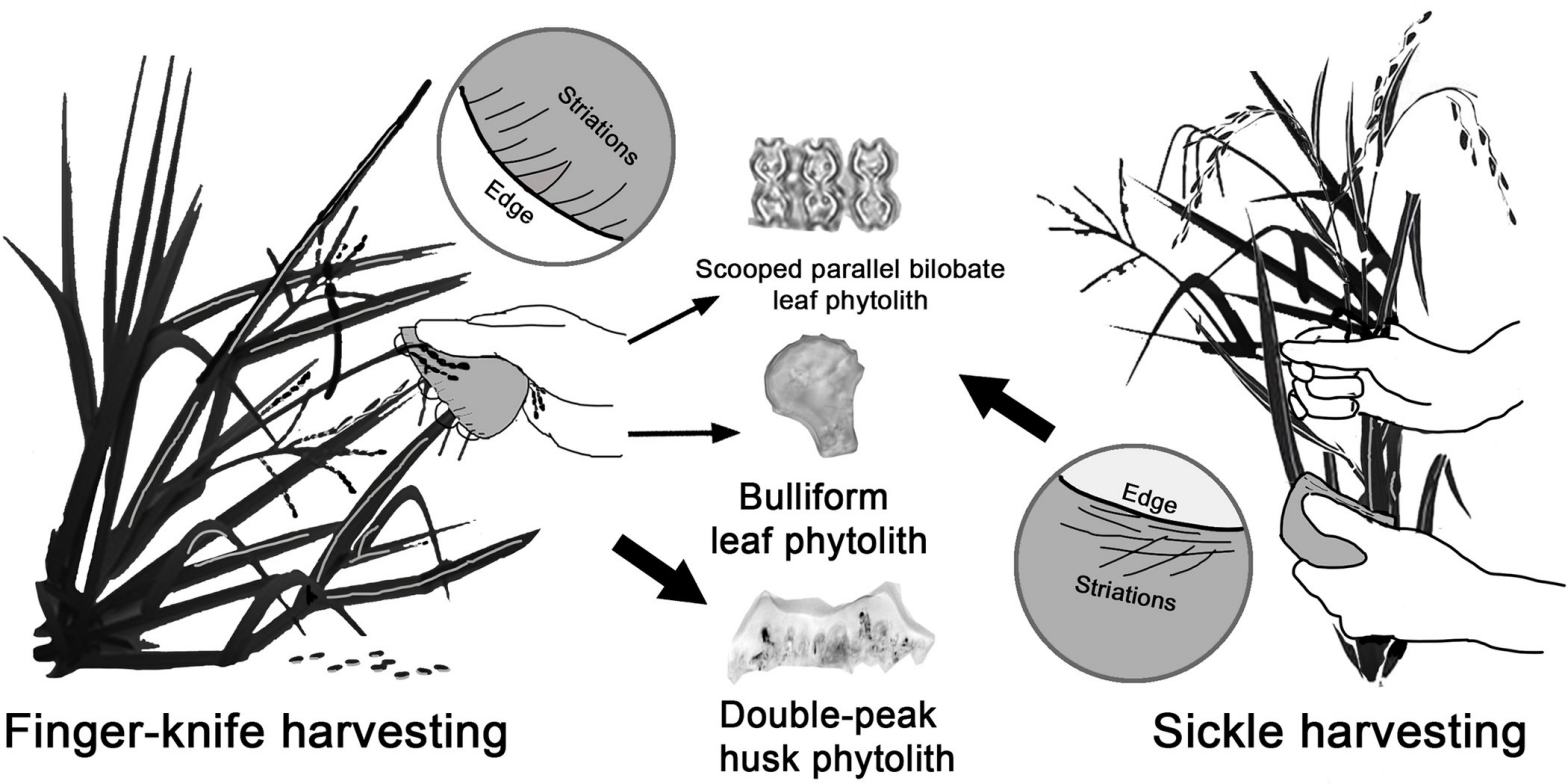
Schematic representation of the use-wear traces and phytoliths from rice-harvesting finger knives and sickles.

**Fig 8:**
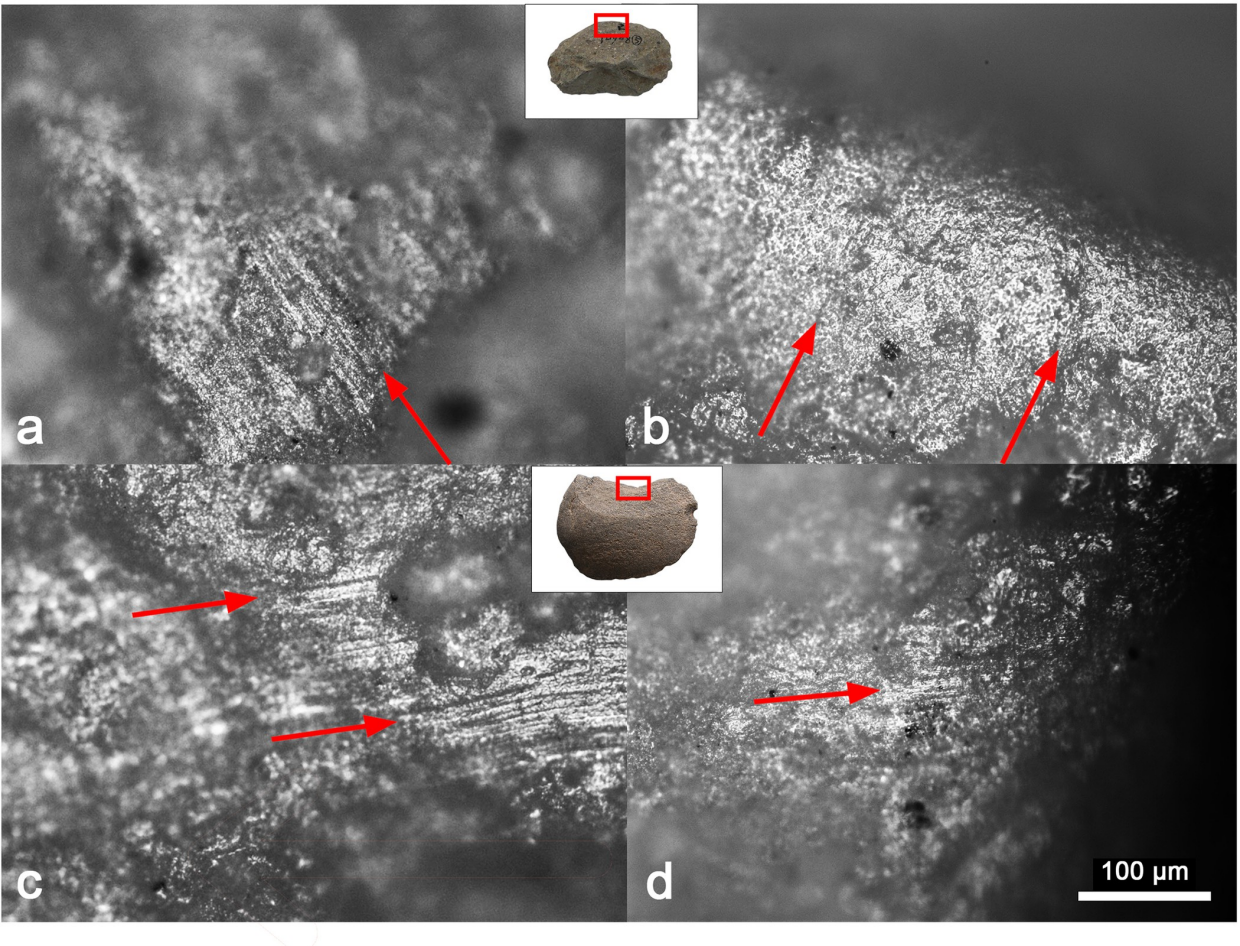
Use-wear traces from rice harvesting flakes. (a) and (b) Use-wear traces from Artifact SS-10 exhibit fine striations perpendicular or diagonal to the cutting edge, suggesting a transverse motion. (c) and (d) Use-wear traces from Artifact HHS-38 are dominated by striations parallel to the cutting edge, suggesting a slicing motion.

Among the 30 flakes that exhibit use-wear traces interpreted to have been produced by plant harvesting, 28 also contain phytolith residues from rice. Furthermore, the integrated use-wear and phytolith data suggest that the flakes have been used for two harvesting modes: reaping the panicles at the top (i.e., finger knife) and cutting the stalk near the base (i.e., sickle) (Fig 7). The finger knife technique is best exemplified by Artifact SS-10. This small flake is made of river pebble, with a slightly convex working edge 58 mm long. Use-wear analysis revealed numerous fine striations perpendicular to the edges, suggesting cutting and/or scraping actions (Fig 8A and 8B). From the tool’s cutting edge, a high proportion of rice glume double-peak phytoliths were recovered, indicating that the tool has been in contact with rice seeds. The presence of perpendicular striations and rice husk phytoliths suggest that the tool was likely used to harvest rice by reaping panicles at the top. In contrast, sickle harvesting generally involves cutting a handful of stalks near the base with a slicing motion. This technique is best demonstrated by Artifact HHS 38, a flake with a concave cutting edge about 92 mm long. The tool exhibits mostly fine and long striations parallel to the cutting edge, suggesting slicing or sawing actions (Fig 8C and 8D). Phytolith residues associated with tool use are predominated by morphotypes from grass leaves and stems, including *Oryza*-type bulliform and scooped parallel bilobate from rice leaf, but no rice husk is present. This pattern indicates that the flake was used as a sickle to cut rice stalks near the ground.

Overall, the results of use-wear and phytolith analyses confirmed our hypothesis, indicating that the early Neolithic people used stone flakes to harvest rice. More specifically, a shift of harvesting techniques likely occurred from the Shangshan to the Kuahuqiao. In the Shangshan, rice was primarily harvested with finger knives, and occasionally, sickles; in the Kuahuqiao, sickle harvesting became the dominant technique (Fig 6B–6D).

## Discussion and conclusion

According to ethnographic records, both finger knife and sickle techniques are traditional to rice farming communities in Asia, and their uses are associated with practical and cultural concerns (for a review, see [34]). Finger knives allow people to selectively harvest individual panicles on multiple occasions over an expanded period, a method well suited for an area where rice does not ripen simultaneously [34–36]. The tool is also effective in reducing shattering during harvest, thus conserving the greatest numbers of grains from the panicle [37]. Sickles, on the other hand, have the capacity to cut a bunch of rice stems at once, thus greatly increasing the speed of harvesting [38]. The harvested rice straws and leaves can be used for fuel and animal feed. The choice of harvesting techniques is also deeply intertwined with spiritual beliefs. In many Southeast Asian communities, finger knives survived long after the introduction of more efficient tools including sickles. Their persistence was partially related to indigenous beliefs that knives protect the “soul of rice” and satisfy the “rice goddess,” thereby providing farmers with abundant harvests [39–41].

While we still know little about the spiritual life of the early Neolithic communities in the Lower Yangtze, we may link their rice harvesting methods to the process of rice domestication. The predominant use of finger knives in the Shangshan culture was likely an adaptation to the rice habitats in the early Holocene. Unlike the wheat and barley in the Near East [42], the wild progenitor of East Asian rice (*Oryza rufipogen*) grows in the swamp wetland, with seeds that ripen unevenly and shatter automatically into muddy water [43]. Harvesting experiments indicate that wild rice–with its roots submerged in the deep-watered wetland–is extremely difficult to be harvested by sickles; instead, using a finger knife to reap rice panicles is more effective [44]. A similar situation likely occurred in the early Holocene, when the process of rice domestication had initiated but stayed at a low level [45]. Analyses of rice spikelet bases from Huxi, a late phase Shangshan culture settlement dating to 9000–8400 cal. BP, indicate that the non-shattering domestic type accounts for only 8.7% of the total [1]. Thus, the Shangshan people likely encountered rice fields dominated by shattering types and uneven ripening. By using a finger knife, they were able to selectively cut near-mature rice panicles from any given stand, leaving the immature panicles behind for the next harvests and thus increasing the total yields [34, 36].

The intensified anthropogenic practices in the Kuahuqiao likely contributed to the increased use of sickles. Around 8000 cal. BP, the Kuahuqiao people had established a system of rice cultivation in grass-reed-swamp environments, with water control and fire management of multiple wetland and forest species [46–48]. Such practices would promote rice to develop more synchronous ripening and erect growth, making sickle harvesting possible. Additional archaeological evidence from the Kuahuqiao culture type-site also lends strong support to this scenario. At this waterlogged settlement, a bundle of rice stalks was found in the residential area at the time of excavation. The stalks were cut off squarely near the base, most likely a result of sickle harvesting [17].

The development of sickle harvesting techniques was a cross-regional practice among the early rice cultivation communities around 8000 BP. At Jiahu, a Peiligang culture site in the Huai River Valley, large quantities of denticulate sickles began to appear in Phase III (8000–7500 BP) [49]. Some of the sickles were used to harvest Poaceae plants including rice, as indicated by use-wear and residue analyses [20, 31, 50]. Phytolith analysis also shows a concurrent increase of rice leaves and stems in Jiahu’s residential areas [51], suggesting that rice stalks were harvested and brought back to the site [52].

The shift from finger knife to sickle harvesting likely led to different post-harvest activities. Harvesting the panicles would allow people to leave the stalks in the field and obtain relatively “clean” harvests with few weeds, whereas cutting the stalks would incorporate more weeds and require additional processing [52–54]. Compared to the Shangshan culture flakes, the Kuahuqiao culture flakes contained more phytoliths from sedges (Cyperaceae), a common weed in rice fields, thus confirming their use as sickles (Fig 6C) [55]. The adoption of sickle harvesting was also accompanied by increased exploitation of reeds (*Phragmites*), which are thick-stemmed aquatic plants coexistent with rice and whose culms are frequently used as raw materials for basketry and matting. Although no item manufactured from reeds has been discovered at Kuahuqiao culture sites yet, reed-woven mats have been found at later Neolithic sites in the same region, including Tianluoshan (ca. 7000 BP) and Hemudu (ca. 6000 BP) [56–58].

Both finger-knife and sickle harvesting would produce unconscious selection for the non-shattering genotype [5]. During the initial stage of rice domestication, the crop’s non-synchronous ripening would encourage multiple episodes of harvests, with later harvests including higher proportions of non-shattering genotypes. If part of the later harvests were saved for sowing, there would be selective pressure for non-shattering [13]. When harvesting rice culms with sickles, some of the matured ears would disarticulate and fall to the ground, increasing the relative proportion of tough-rachised ears in the harvested population. Stimulation data suggest that sickle harvesting could induce rapid selection of the tough rachis mutant, complete within two centuries [5]. Archaeobotanical data, however, indicate a much slower process of rice domestication that lasted for almost five thousand years [3, 4]. The protracted process of domestication was likely related to practical and cultural factors. Without scientific knowledge, the incipient rice cultivators might not store late harvests for sowing, use virgin plots to sow every year, or keep sufficient distances between domesticated and wild stands—all these practices would make the actual domestication much slower than what is predicted under ideal circumstances. At the same time, the initial motivation for intensive rice cultivation probably remained at a low level for an extended period, considering the abundant wild food resources in the Lower Yangtze. At Kuahuqiao (8000–7000 BP), after at least 2000 years of cultivation, rice only constituted a small component of the human diet; wild plants, such as acorns, were still the staple foods [28]. Without resource pressure, the incipient rice cultivation was likely a supplementary activity, without much expectation for return.

After 7000 BP, rice cultivation continued throughout the subsequent Hemudu culture and eventually became fully domesticated in the Liangzhu culture (5300–4400 BP)—southern China’s first state-level society [4, 59]. Large quantities of knife-shaped stone tools (*daoxingqi*), possibly hafted, have been recovered from the middle and late Neolithic settlements [58, 60, 61]. However, their functions remain unclear due to the lack of scientific analyses. Future research on these tools is needed to evaluate plant harvesting techniques, hafting arrangements, and cultivation intensity during the later stages of agricultural transition.

## Supporting information

S1 Table Use-wear data. (XLSX)

S2 Table Phytolith residue data. (XLSX)
