# Supplementary material for: New evidence for rice harvesting in the early Neolithic Lower Yangtze River, China
Source: PLoS One. 2022 Dec 7;17(12):e0278200. doi: 10.1371/journal.pone.0278200 (PMC9728911; doi:10.1371/journal.pone.0278200)
Supplement: Attachment — Submitted filename: Review of Wang et al PLOS One 2022.docx [file pone.0278200.s003.docx]

**Review of:** Wang *et al* **– ‘**New evidence for rice harvesting in the early Neolithic Lower Yangtze River, China’

**Overall review**

- Conceptually I think the paper is very interesting, the integration of use-wear data (which is treated with the interpretative care that we alas often do not see), and phytolith analyses producing very convincing, and thoughtful claims and discussion.

**General comments**

- My background in microwear analysis makes me strongly request that the word ‘diagnostic’ in line 59, is replaced with ‘distinctive’ (there are other silica rich plants aside from cereals that will produce lustrous surfaces, e.g., materials for basketry).
- For the general reader, I think the paper would benefit from a figure, or table that sketches out neatly the culture groups, chronology, and position within the rice cultivation spectrum.
- Line 107 – can you state what magnification you were using the DinoLite microscope for the sampling process.
- I am very happy with how the use-wear study has been dealt with; the authors are to be applauded for their interpretative honesty, i.e., that the some of the ‘polishes’ relate to silica-rich plant use (though ‘plants’, not ‘lants’ in line 154), which could be rice, matting, basketry etc. (the common use wear patterns of exploiting silica rich plants (Poaceae) that they have investigated experimentally). It is only with the integration of the phytolith data, that they suggest that the use-wear relates to harvesting rice – bravo!
- Honestly, having read so many use-wear papers where practitioners claim to recognise media-specific polishes, this paper makes me *very* happy as it shows how we can – and should – productively use this form of analysis.
