# Supplementary material for: New evidence for rice harvesting in the early Neolithic Lower Yangtze River, China
Source: PLoS One. 2022 Dec 7;17(12):e0278200. doi: 10.1371/journal.pone.0278200 (PMC9728911; doi:10.1371/journal.pone.0278200)
Supplement: Attachment — Submitted filename: Response to reviewers.docx [file pone.0278200.s004.docx]

**Response to reviewers**

When submitting your revision, we need you to address these additional requirements.

1. Please ensure that your manuscript meets PLOS ONE's style requirements, including those for file naming. The PLOS ONE style templates can be found at 
<https://journals.plos.org/plosone/s/file?id=wjVg/PLOSOne_formatting_sample_main_body.pdf> and 
<https://journals.plos.org/plosone/s/file?id=ba62/PLOSOne_formatting_sample_title_authors_affiliations.pdf>

Response:

We have modified the manuscript by following PLOS ONE’s style requirements.

2. We note that the grant information you provided in the ‘Funding Information’ and ‘Financial Disclosure’ sections do not match.

When you resubmit, please ensure that you provide the correct grant numbers for the awards you received for your study in the ‘Funding Information’ section.

Response:

We have modified the grant information in the system.

3. We note that Figure 1 in your submission contain [map/satellite] images which may be copyrighted. All PLOS content is published under the Creative Commons Attribution License (CC BY 4.0), which means that the manuscript, images, and Supporting Information files will be freely available online, and any third party is permitted to access, download, copy, distribute, and use these materials in any way, even commercially, with proper attribution. For these reasons, we cannot publish previously copyrighted maps or satellite images created using proprietary data, such as Google software (Google Maps, Street View, and Earth). For more information, see our copyright guidelines: <http://journals.plos.org/plosone/s/licenses-and-copyright>.
 
We require you to either (a) present written permission from the copyright holder to publish these figures specifically under the CC BY 4.0 license, or (b) remove the figures from your submission:
 
a. You may seek permission from the original copyright holder of Figure 1 to publish the content specifically under the CC BY 4.0 license.  
 
We recommend that you contact the original copyright holder with the Content Permission Form (<http://journals.plos.org/plosone/s/file?id=7c09/content-permission-form.pdf>) and the following text:
“I request permission for the open-access journal PLOS ONE to publish XXX under the Creative Commons Attribution License (CCAL) CC BY 4.0 (<http://creativecommons.org/licenses/by/4.0/>). Please be aware that this license allows unrestricted use and distribution, even commercially, by third parties. Please reply and provide explicit written permission to publish XXX under a CC BY license and complete the attached form.”
 
Please upload the completed Content Permission Form or other proof of granted permissions as an "Other" file with your submission.
 
In the figure caption of the copyrighted figure, please include the following text: “Reprinted from [ref] under a CC BY license, with permission from [name of publisher], original copyright [original copyright year].”
 
b. If you are unable to obtain permission from the original copyright holder to publish these figures under the CC BY 4.0 license or if the copyright holder’s requirements are incompatible with the CC BY 4.0 license, please either i) remove the figure or ii) supply a replacement figure that complies with the CC BY 4.0 license. Please check copyright information on all replacement figures and update the figure caption with source information. If applicable, please specify in the figure caption text when a figure is similar but not identical to the original image and is therefore for illustrative purposes only.
The following resources for replacing copyrighted map figures may be helpful:
 
USGS National Map Viewer (public domain): <http://viewer.nationalmap.gov/viewer/>
The Gateway to Astronaut Photography of Earth (public domain): <http://eol.jsc.nasa.gov/sseop/clickmap/>
Maps at the CIA (public domain): <https://www.cia.gov/library/publications/the-world-factbook/index.html> and <https://www.cia.gov/library/publications/cia-maps-publications/index.html>
NASA Earth Observatory (public domain): <http://earthobservatory.nasa.gov/>
Landsat: <http://landsat.visibleearth.nasa.gov/>
USGS EROS (Earth Resources Observatory and Science (EROS) Center) (public domain): [http://eros.usgs.gov/#](http://eros.usgs.gov/)
Natural Earth (public domain): <http://www.naturalearthdata.com/>

Response:

The current Figure is based on a map from USGS National Map viewer, so it is not copy righted. We have added this information in the figure caption text.

4.Please review your reference list to ensure that it is complete and correct. If you have cited papers that have been retracted, please include the rationale for doing so in the manuscript text, or remove these references and replace them with relevant current references. Any changes to the reference list should be mentioned in the rebuttal letter that accompanies your revised manuscript. If you need to cite a retracted article, indicate the article’s retracted status in the References list and also include a citation and full reference for the retraction notice.

Response:

We have checked our reference list.

Reviewers' comments:

Reviewer #1: This is a delightful paper on harvesting techniques and their biological implications in the lower Yangtze River, based on use-wear and phytolith evidence from 52 flaked stone tools from Shangshan and Hehuashan cultural sites, dating between 10k and 7k BP. It demonstrates a clear and interesting trend in shifting traditions from using finger-knife to cut plant panicles to sickle harvesting at relatively lower positions of the plants. It relates the former with the asynchronous nature of wild rice ripening and the necessity of multiple episodes of harvesting events and the latter with the selective advantage of non-shattering grains. The discussion of the implication of the slower process of rice domestication and consciousness towards the end of the paper is excellent and constitutes a significant contribution. Additional consideration of the consequences of lower harvesting (sickle) to weed taxonomy in archaeobotanical assemblage will add additional value (cf. G. Jones 1984, originally and subsequent literature). This article contributes substantively to a series of research concerning crop processing in East Asia, additional reference to Liu et al. 2017 on social context underlining harvesting/processing techniques will be helpful. I think this should be published.


JONES, G.E.M. 1984. Interpretation of plant remains: ethnographic models from Greece, in W. VAN ZEIST & W.A. CASPARIE (eds.) Plants and Ancient Man, Studies in Palaeoethnobotany: proceedings of the 6th symposium of the international work group for palaeoethnobotany: 43-61. Groningen: A. A. Balkema.

LIU, X., ZHAO, Z. & JONES, M.K. 2017. From people's commune to household responsibility: Ethnoarchaeological perspectives of millet production in prehistoric northeast China. Archaeological Research in Asia 11: 51-57.

Response:

Thank you for your comments. We have added your recommended reference and related discussions.

Reviewer #2: Overall review
• Conceptually I think the paper is very interesting, the integration of use-wear data (which is treated with the interpretative care that we alas often do not see), and phytolith analyses producing very convincing, and thoughtful claims and discussion.

General comments
• My background in microwear analysis makes me strongly request that the word ‘diagnostic’ in line 59, is replaced with ‘distinctive’ (there are other silica rich plants aside from cereals that will produce lustrous surfaces, e.g., materials for basketry).

Response:

Thanks for your suggestion – we have replaced “diagnostic” with “distinctive”.

• For the general reader, I think the paper would benefit from a figure, or table that sketches out neatly the culture groups, chronology, and position within the rice cultivation spectrum.

Response:

We have added a graph in Figure 1 to show the chronology, cultural groups, and rice domestication process in the study region.

• Line 107 – can you state what magnification you were using the DinoLite microscope for the sampling process.

Response:

The magnification is 10× to 50×, and we have added the information in the revised manuscript.

• I am very happy with how the use-wear study has been dealt with; the authors are to be applauded for their interpretative honesty, i.e., that the some of the ‘polishes’ relate to silica-rich plant use (though ‘plants’, not ‘lants’ in line 154), which could be rice, matting, basketry etc. (the common use wear patterns of exploiting silica rich plants (Poaceae) that they have investigated experimentally). It is only with the integration of the phytolith data, that they suggest that the use-wear relates to harvesting rice – bravo!

Response:

Thank you. We have corrected the error in line 154.

• Honestly, having read so many use-wear papers where practitioners claim to recognise media-specific polishes, this paper makes me very happy as it shows how we can – and should – productively use this form of analysis.

Response: Thank you!

Reviewer #3: This is a very interesting paper! The authors were able to reveal the functionalities of the stone tools from two early farming societies in southern China, providing valuable information to understand the early technological choices for harvesting rice. Here I propose two opinions that the authors may consider. Firstly, as suggested by the use-wear analysis, some of the stone tools were not only associated with rice harvesting but also involved in processing animal tissues and wood. These findings support the multifunctional roles of these stone assemblages. However, this point has not been discussed or mentioned in the text much.

Response:

Thank you for your suggestion. We have added a paragraph to discuss multifunctional use of tools.

Secondly, the statement starting from line 34-“Most studies….” seems to contradict the following sentences starting from line 38- “Harvesting functions….”. Apart from these two minor issues, I barely have any other suggestions for this article and thus recommend “Minor Revision” for it.

Response:

Thank you for your comments. We have revised the first statement to clarify the logical connection. Now the first statement reads as:

Most previous studies have focused on analyzing morphological changes of rice remains to trace the timeline of rice domestication, whereas few scholars have paid attention to the cultivation practices that gave rise to domesticated forms.
