# Supplementary material for: New evidence for rice harvesting in the early Neolithic Lower Yangtze River, China
Source: PLoS One. 2022 Dec 7;17(12):e0278200. doi: 10.1371/journal.pone.0278200 (PMC9728911; doi:10.1371/journal.pone.0278200)
Supplement: Peer review history

# Peer review: Decision Letter 0

**DOI:** 10.1371/journal.pone.0278200.r001

**Authors:** Peter F. Biehl

6 Nov 2022

PONE-D-22-25920New evidence for rice harvesting in the early Neolithic Lower Yangtze River, ChinaPLOS ONE

Dear Dr. Wang,

Thank you for submitting your manuscript to PLOS ONE. After careful consideration, we feel that it has merit but does not fully meet PLOS ONE’s publication criteria as it currently stands. Therefore, we invite you to submit a revised version of the manuscript that addresses the points raised during the review process.

Please submit your revised manuscript by Dec 21 2022 11:59PM. If you will need more time than this to complete your revisions, please reply to this message or contact the journal office at plosone@plos.org. When you're ready to submit your revision, log on to https://www.editorialmanager.com/pone/ and select the 'Submissions Needing Revision' folder to locate your manuscript file.

Please include the following items when submitting your revised manuscript:

- A rebuttal letter that responds to each point raised by the academic editor and reviewer(s). You should upload this letter as a separate file labeled 'Response to Reviewers'.
- A marked-up copy of your manuscript that highlights changes made to the original version. You should upload this as a separate file labeled 'Revised Manuscript with Track Changes'.
- An unmarked version of your revised paper without tracked changes. You should upload this as a separate file labeled 'Manuscript'.

If you would like to make changes to your financial disclosure, please include your updated statement in your cover letter. Guidelines for resubmitting your figure files are available below the reviewer comments at the end of this letter.

If applicable, we recommend that you deposit your laboratory protocols in protocols.io to enhance the reproducibility of your results. Protocols.io assigns your protocol its own identifier (DOI) so that it can be cited independently in the future. For instructions see: https://journals.plos.org/plosone/s/submission-guidelines#loc-laboratory-protocols. Additionally, PLOS ONE offers an option for publishing peer-reviewed Lab Protocol articles, which describe protocols hosted on protocols.io. Read more information on sharing protocols at https://plos.org/protocols?utm_medium=editorial-email&utm_source=authorletters&utm_campaign=protocols.

We look forward to receiving your revised manuscript.

Kind regards,

Peter F. Biehl, PhD

Academic Editor

PLOS ONE

Journal Requirements:

When submitting your revision, we need you to address these additional requirements.

1. Please ensure that your manuscript meets PLOS ONE's style requirements, including those for file naming. The PLOS ONE style templates can be found at 

https://journals.plos.org/plosone/s/file?id=wjVg/PLOSOne_formatting_sample_main_body.pdf and 

https://journals.plos.org/plosone/s/file?id=ba62/PLOSOne_formatting_sample_title_authors_affiliations.pdf

2. We note that the grant information you provided in the ‘Funding Information’ and ‘Financial Disclosure’ sections do not match. 

When you resubmit, please ensure that you provide the correct grant numbers for the awards you received for your study in the ‘Funding Information’ section.

3. We note that Figure 1 in your submission contain [map/satellite] images which may be copyrighted. All PLOS content is published under the Creative Commons Attribution License (CC BY 4.0), which means that the manuscript, images, and Supporting Information files will be freely available online, and any third party is permitted to access, download, copy, distribute, and use these materials in any way, even commercially, with proper attribution. For these reasons, we cannot publish previously copyrighted maps or satellite images created using proprietary data, such as Google software (Google Maps, Street View, and Earth). For more information, see our copyright guidelines: http://journals.plos.org/plosone/s/licenses-and-copyright.

We require you to either (a) present written permission from the copyright holder to publish these figures specifically under the CC BY 4.0 license, or (b) remove the figures from your submission:

a. You may seek permission from the original copyright holder of Figure 1 to publish the content specifically under the CC BY 4.0 license.  

We recommend that you contact the original copyright holder with the Content Permission Form (http://journals.plos.org/plosone/s/file?id=7c09/content-permission-form.pdf) and the following text:

“I request permission for the open-access journal PLOS ONE to publish XXX under the Creative Commons Attribution License (CCAL) CC BY 4.0 (http://creativecommons.org/licenses/by/4.0/). Please be aware that this license allows unrestricted use and distribution, even commercially, by third parties. Please reply and provide explicit written permission to publish XXX under a CC BY license and complete the attached form.”

Please upload the completed Content Permission Form or other proof of granted permissions as an "Other" file with your submission.

In the figure caption of the copyrighted figure, please include the following text: “Reprinted from [ref] under a CC BY license, with permission from [name of publisher], original copyright [original copyright year].”

b. If you are unable to obtain permission from the original copyright holder to publish these figures under the CC BY 4.0 license or if the copyright holder’s requirements are incompatible with the CC BY 4.0 license, please either i) remove the figure or ii) supply a replacement figure that complies with the CC BY 4.0 license. Please check copyright information on all replacement figures and update the figure caption with source information. If applicable, please specify in the figure caption text when a figure is similar but not identical to the original image and is therefore for illustrative purposes only.

The following resources for replacing copyrighted map figures may be helpful:

USGS National Map Viewer (public domain): http://viewer.nationalmap.gov/viewer/

The Gateway to Astronaut Photography of Earth (public domain): http://eol.jsc.nasa.gov/sseop/clickmap/

Maps at the CIA (public domain): https://www.cia.gov/library/publications/the-world-factbook/index.html and https://www.cia.gov/library/publications/cia-maps-publications/index.html

NASA Earth Observatory (public domain): http://earthobservatory.nasa.gov/

Landsat: http://landsat.visibleearth.nasa.gov/

USGS EROS (Earth Resources Observatory and Science (EROS) Center) (public domain): http://eros.usgs.gov/#

Natural Earth (public domain): http://www.naturalearthdata.com/

4.Please review your reference list to ensure that it is complete and correct. If you have cited papers that have been retracted, please include the rationale for doing so in the manuscript text, or remove these references and replace them with relevant current references. Any changes to the reference list should be mentioned in the rebuttal letter that accompanies your revised manuscript. If you need to cite a retracted article, indicate the article’s retracted status in the References list and also include a citation and full reference for the retraction notice.

Additional Editor Comments:

Please address the minor revisions indicated by the reviewers and re-submit.

[Note: HTML markup is below. Please do not edit.]

Reviewers' comments:

Reviewer's Responses to Questions

**Comments to the Author**

1. Is the manuscript technically sound, and do the data support the conclusions?

The manuscript must describe a technically sound piece of scientific research with data that supports the conclusions. Experiments must have been conducted rigorously, with appropriate controls, replication, and sample sizes. The conclusions must be drawn appropriately based on the data presented. 

Reviewer #1: Yes

Reviewer #2: Yes

Reviewer #3: Yes

**********

2. Has the statistical analysis been performed appropriately and rigorously? 

Reviewer #1: N/A

Reviewer #2: I Don't Know

Reviewer #3: Yes

**********

3. Have the authors made all data underlying the findings in their manuscript fully available?

The PLOS Data policy requires authors to make all data underlying the findings described in their manuscript fully available without restriction, with rare exception (please refer to the Data Availability Statement in the manuscript PDF file). The data should be provided as part of the manuscript or its supporting information, or deposited to a public repository. For example, in addition to summary statistics, the data points behind means, medians and variance measures should be available. If there are restrictions on publicly sharing data—e.g. participant privacy or use of data from a third party—those must be specified.

Reviewer #1: Yes

Reviewer #2: Yes

Reviewer #3: Yes

**********

4. Is the manuscript presented in an intelligible fashion and written in standard English?

PLOS ONE does not copyedit accepted manuscripts, so the language in submitted articles must be clear, correct, and unambiguous. Any typographical or grammatical errors should be corrected at revision, so please note any specific errors here.

Reviewer #1: Yes

Reviewer #2: Yes

Reviewer #3: Yes

**********

5. Review Comments to the Author

Please use the space provided to explain your answers to the questions above. You may also include additional comments for the author, including concerns about dual publication, research ethics, or publication ethics. (Please upload your review as an attachment if it exceeds 20,000 characters)

Reviewer #1: This is a delightful paper on harvesting techniques and their biological implications in the lower Yangtze River, based on use-wear and phytolith evidence from 52 flaked stone tools from Shangshan and Hehuashan cultural sites, dating between 10k and 7k BP. It demonstrates a clear and interesting trend in shifting traditions from using finger-knife to cut plant panicles to sickle harvesting at relatively lower positions of the plants. It relates the former with the asynchronous nature of wild rice ripening and the necessity of multiple episodes of harvesting events and the latter with the selective advantage of non-shattering grains. The discussion of the implication of the slower process of rice domestication and consciousness towards the end of the paper is excellent and constitutes a significant contribution. Additional consideration of the consequences of lower harvesting (sickle) to weed taxonomy in archaeobotanical assemblage will add additional value (cf. G. Jones 1984, originally and subsequent literature). This article contributes substantively to a series of research concerning crop processing in East Asia, additional reference to Liu et al. 2017 on social context underlining harvesting/processing techniques will be helpful. I think this should be published.

JONES, G.E.M. 1984. Interpretation of plant remains: ethnographic models from Greece, in W. VAN ZEIST & W.A. CASPARIE (eds.) Plants and Ancient Man, Studies in Palaeoethnobotany: proceedings of the 6th symposium of the international work group for palaeoethnobotany: 43-61. Groningen: A. A. Balkema.

LIU, X., ZHAO, Z. & JONES, M.K. 2017. From people's commune to household responsibility: Ethnoarchaeological perspectives of millet production in prehistoric northeast China. Archaeological Research in Asia 11: 51-57.

Reviewer #2: Overall review

• Conceptually I think the paper is very interesting, the integration of use-wear data (which is treated with the interpretative care that we alas often do not see), and phytolith analyses producing very convincing, and thoughtful claims and discussion.

General comments

• My background in microwear analysis makes me strongly request that the word ‘diagnostic’ in line 59, is replaced with ‘distinctive’ (there are other silica rich plants aside from cereals that will produce lustrous surfaces, e.g., materials for basketry).

• For the general reader, I think the paper would benefit from a figure, or table that sketches out neatly the culture groups, chronology, and position within the rice cultivation spectrum.

• Line 107 – can you state what magnification you were using the DinoLite microscope for the sampling process.

• I am very happy with how the use-wear study has been dealt with; the authors are to be applauded for their interpretative honesty, i.e., that the some of the ‘polishes’ relate to silica-rich plant use (though ‘plants’, not ‘lants’ in line 154), which could be rice, matting, basketry etc. (the common use wear patterns of exploiting silica rich plants (Poaceae) that they have investigated experimentally). It is only with the integration of the phytolith data, that they suggest that the use-wear relates to harvesting rice – bravo!

• Honestly, having read so many use-wear papers where practitioners claim to recognise media-specific polishes, this paper makes me very happy as it shows how we can – and should – productively use this form of analysis.

Reviewer #3: This is a very interesting paper! The authors were able to reveal the functionalities of the stone tools from two early farming societies in southern China, providing valuable information to understand the early technological choices for harvesting rice. Here I propose two opinions that the authors may consider. Firstly, as suggested by the use-wear analysis, some of the stone tools were not only associated with rice harvesting but also involved in processing animal tissues and wood. These findings support the multifunctional roles of these stone assemblages. However, this point has not been discussed or mentioned in the text much. Secondly, the statement starting from line 34-“Most studies….” seems to contradict the following sentences starting from line 38- “Harvesting functions….”. Apart from these two minor issues, I barely have any other suggestions for this article and thus recommend “Minor Revision” for it.

**********

6. PLOS authors have the option to publish the peer review history of their article (what does this mean?). If published, this will include your full peer review and any attached files.

If you choose “no”, your identity will remain anonymous but your review may still be made public.

**Do you want your identity to be public for this peer review?** For information about this choice, including consent withdrawal, please see our Privacy Policy.

Reviewer #1: No

Reviewer #2: **Yes: **Tristan Carter

Reviewer #3: No

**********

[NOTE: If reviewer comments were submitted as an attachment file, they will be attached to this email and accessible via the submission site. Please log into your account, locate the manuscript record, and check for the action link "View Attachments". If this link does not appear, there are no attachment files.]

While revising your submission, please upload your figure files to the Preflight Analysis and Conversion Engine (PACE) digital diagnostic tool, https://pacev2.apexcovantage.com/. PACE helps ensure that figures meet PLOS requirements. To use PACE, you must first register as a user. Registration is free. Then, login and navigate to the UPLOAD tab, where you will find detailed instructions on how to use the tool. If you encounter any issues or have any questions when using PACE, please email PLOS at figures@plos.org. Please note that Supporting Information files do not need this step.

Attachment Submitted filename: Review of Wang et al PLOS One 2022.docx Click here for additional data file.

# Author comment: Author response to Decision Letter 0

**DOI:** 10.1371/journal.pone.0278200.r002

9 Nov 2022

Reviewers' comments:

Reviewer #1: This is a delightful paper on harvesting techniques and their biological implications in the lower Yangtze River, based on use-wear and phytolith evidence from 52 flaked stone tools from Shangshan and Hehuashan cultural sites, dating between 10k and 7k BP. It demonstrates a clear and interesting trend in shifting traditions from using finger-knife to cut plant panicles to sickle harvesting at relatively lower positions of the plants. It relates the former with the asynchronous nature of wild rice ripening and the necessity of multiple episodes of harvesting events and the latter with the selective advantage of non-shattering grains. The discussion of the implication of the slower process of rice domestication and consciousness towards the end of the paper is excellent and constitutes a significant contribution. Additional consideration of the consequences of lower harvesting (sickle) to weed taxonomy in archaeobotanical assemblage will add additional value (cf. G. Jones 1984, originally and subsequent literature). This article contributes substantively to a series of research concerning crop processing in East Asia, additional reference to Liu et al. 2017 on social context underlining harvesting/processing techniques will be helpful. I think this should be published.

JONES, G.E.M. 1984. Interpretation of plant remains: ethnographic models from Greece, in W. VAN ZEIST & W.A. CASPARIE (eds.) Plants and Ancient Man, Studies in Palaeoethnobotany: proceedings of the 6th symposium of the international work group for palaeoethnobotany: 43-61. Groningen: A. A. Balkema.

LIU, X., ZHAO, Z. & JONES, M.K. 2017. From people's commune to household responsibility: Ethnoarchaeological perspectives of millet production in prehistoric northeast China. Archaeological Research in Asia 11: 51-57.

Response:

Thank you for your comments. We have added your recommended reference and related discussions. 

Reviewer #2: Overall review

• Conceptually I think the paper is very interesting, the integration of use-wear data (which is treated with the interpretative care that we alas often do not see), and phytolith analyses producing very convincing, and thoughtful claims and discussion.

General comments

• My background in microwear analysis makes me strongly request that the word ‘diagnostic’ in line 59, is replaced with ‘distinctive’ (there are other silica rich plants aside from cereals that will produce lustrous surfaces, e.g., materials for basketry).

Response:

Thanks for your suggestion – we have replaced “diagnostic” with “distinctive”.

• For the general reader, I think the paper would benefit from a figure, or table that sketches out neatly the culture groups, chronology, and position within the rice cultivation spectrum.

Response:

We have added a graph in Figure 1 to show the chronology, cultural groups, and rice domestication process in the study region. 

• Line 107 – can you state what magnification you were using the DinoLite microscope for the sampling process.

Response: 

The magnification is 10× to 50×, and we have added the information in the revised manuscript. 

• I am very happy with how the use-wear study has been dealt with; the authors are to be applauded for their interpretative honesty, i.e., that the some of the ‘polishes’ relate to silica-rich plant use (though ‘plants’, not ‘lants’ in line 154), which could be rice, matting, basketry etc. (the common use wear patterns of exploiting silica rich plants (Poaceae) that they have investigated experimentally). It is only with the integration of the phytolith data, that they suggest that the use-wear relates to harvesting rice – bravo!

Response: 

Thank you. We have corrected the error in line 154. 

• Honestly, having read so many use-wear papers where practitioners claim to recognise media-specific polishes, this paper makes me very happy as it shows how we can – and should – productively use this form of analysis.

Response: Thank you!

Reviewer #3: This is a very interesting paper! The authors were able to reveal the functionalities of the stone tools from two early farming societies in southern China, providing valuable information to understand the early technological choices for harvesting rice. Here I propose two opinions that the authors may consider. Firstly, as suggested by the use-wear analysis, some of the stone tools were not only associated with rice harvesting but also involved in processing animal tissues and wood. These findings support the multifunctional roles of these stone assemblages. However, this point has not been discussed or mentioned in the text much. 

Response:

Thank you for your suggestion. We have added a paragraph to discuss multifunctional use of tools. 

Secondly, the statement starting from line 34-“Most studies….” seems to contradict the following sentences starting from line 38- “Harvesting functions….”. Apart from these two minor issues, I barely have any other suggestions for this article and thus recommend “Minor Revision” for it.

Response:

Thank you for your comments. We have revised the first statement to clarify the logical connection. Now the first statement reads as:

Most previous studies have focused on analyzing morphological changes of rice remains to trace the timeline of rice domestication, whereas few scholars have paid attention to the cultivation practices that gave rise to domesticated forms.

Attachment Submitted filename: Response to reviewers.docx Click here for additional data file.

# Editor report: Decision Letter 1

**DOI:** 10.1371/journal.pone.0278200.r003

**Authors:** Peter F. Biehl

14 Nov 2022

New evidence for rice harvesting in the early Neolithic Lower Yangtze River, China

PONE-D-22-25920R1

Dear Dr. Wang,

We’re pleased to inform you that your manuscript has been judged scientifically suitable for publication and will be formally accepted for publication once it meets all outstanding technical requirements.

Within one week, you’ll receive an e-mail detailing the required amendments. When these have been addressed, you’ll receive a formal acceptance letter and your manuscript will be scheduled for publication.

An invoice for payment will follow shortly after the formal acceptance. To ensure an efficient process, please log into Editorial Manager at http://www.editorialmanager.com/pone/, click the 'Update My Information' link at the top of the page, and double check that your user information is up-to-date. If you have any billing related questions, please contact our Author Billing department directly at authorbilling@plos.org.

If your institution or institutions have a press office, please notify them about your upcoming paper to help maximize its impact. If they’ll be preparing press materials, please inform our press team as soon as possible -- no later than 48 hours after receiving the formal acceptance. Your manuscript will remain under strict press embargo until 2 pm Eastern Time on the date of publication. For more information, please contact onepress@plos.org.

Kind regards,

Peter F. Biehl, PhD

Academic Editor

PLOS ONE

Additional Editor Comments (optional):

Reviewers' comments:

# Editor report: Acceptance letter

**DOI:** 10.1371/journal.pone.0278200.r004

**Authors:** Peter F. Biehl

15 Nov 2022

PONE-D-22-25920R1 

New evidence for rice harvesting in the early Neolithic Lower Yangtze River, China 

Dear Dr. Wang:

I'm pleased to inform you that your manuscript has been deemed suitable for publication in PLOS ONE. Congratulations! Your manuscript is now with our production department. 

If your institution or institutions have a press office, please let them know about your upcoming paper now to help maximize its impact. If they'll be preparing press materials, please inform our press team within the next 48 hours. Your manuscript will remain under strict press embargo until 2 pm Eastern Time on the date of publication. For more information please contact onepress@plos.org.

If we can help with anything else, please email us at plosone@plos.org. 

Thank you for submitting your work to PLOS ONE and supporting open access. 

Kind regards, 

PLOS ONE Editorial Office Staff

on behalf of

Dr. Peter F. Biehl 

Academic Editor

PLOS ONE
